# Validation of the DigCompThExO questionnaire for assessing exercise therapists’ competences in planning and implementing digital obesity therapy

**DOI:** 10.3389/fspor.2026.1717642

**Published:** 2026-02-09

**Authors:** Sabine Pawellek, Thomas Wendeborn, Hagen Wulff

**Affiliations:** Department of Sport Pedagogy, Faculty of Sport Science, Leipzig University, Leipzig, Germany

**Keywords:** competence model, curriculum development, digital transformation, exercise therapy, obesity

## Abstract

**Background:**

The digital transformation of obesity therapy introduces new demands on exercise therapists. Yet, the therefore required competences remain mostly unassessed, hindering effective implementation. To address this gap, valid instruments are needed to identify competences to plan and implement digital obesity exercise therapy and inform targeted professional training programs. The DigCompThExO model provides a conceptual framework and accompanying questionnaire but has not yet been validated. Therefore, this study examines the factorial structure and psychometric properties to enable its diagnostic use.

**Methods:**

To address the research gap, two consecutive studies were conducted. Study 1 assessed content validity through cognitive interviews with German-speaking digital obesity exercise therapists, using Tourangeau-based probing. Study 2 evaluated structural validity via confirmatory factor analysis (CFA) based on revised questionnaire data from a separate sample within the same target group. Model fit (CFI, TLI, RMSEA, SRMR), validity (AVE, HTMT), reliability (CR), and measurement invariance were evaluated.

**Results:**

Study 1 involved ten therapists (Age: M = 39.3, SD = 10.67, 50% female) in cognitive interviews. Based on results, one dimension and one item were removed; scaling and wording were refined. In Study 2, 205 therapists (Age: M = 32.34, SD = 5.9, 47.3% female, 0.5% divers) completed the revised questionnaire. CFA revealed four latent factors, with good model fit [CFI = .964; TLI = .953; RMSEA = .070, 90% CI (.049;.090); SRMR = .051]. Regarding validity and reliability, measurement invariance was confirmed (RMSEA < .069), along with convergent validity (AVE > .50), discriminant validity (HTMT < .85), and internal consistencies (all CR > .70).

**Conclusion:**

Cognitive interviews and CFA confirm reliability, construct validity, and practical relevance of the DigCompThExO for digital obesity exercise therapy. It enables the assessment of existing and required digital competences and may guide the development of tailored training programs. If embedded in institutional medical education (e.g., university), this could enhance quality and effectiveness of digital obesity exercise therapy. Further research should explore the tool's applicability in other domains of obesity therapy (e.g., nutrition, behavior) or chronic conditions (e.g., diabetes) to promote digital, location-independent healthcare.

## 1 Introduction

Obesity, a multifactorial chronic disease marked by excessive body fat accumulation, is a major global public health issue (1). Projections estimate that by 2030, over one billion people worldwide will be affected (2). Beyond its immediate health impact, obesity is associated with comorbidities (e.g., type 2 diabetes), imposing significant burdens on individuals and healthcare systems (3)*.* Despite evidence-based therapeutic approaches, obesity therapy often fails due to structural barriers including long travel distances, limited local services, and restricted access to specialized treatment centers (4).

Given this problem, digital therapy formats have emerged as a promising solution. They offer time- and location-independent access, reducing infrastructural barriers, and promoting equitable healthcare delivery (5, 6). In line with definitions of digital therapeutics, digital therapy in this study refers to therapeutic interventions delivered via clinically informed, patient-directed digital applications (e.g., video platforms, web-based tools, or mobile software) aimed at supporting the treatment, management, or prevention of disease (7). While initial concepts for digital obesity therapy exist, implementation remains limited. This is partly due to the absence of validated and clearly defined digital competences among therapists (8). Obesity therapy involves a multidisciplinary team of nutritionists, pediatricians, psychologists, and exercise therapists. Although digitalization affects all professional groups, exercise therapists warrant particular attention. Given the pivotal role of exercise in long-term weight management (9), German therapy guidelines assign it as many sessions as the other therapy components (nutrition, behavior, medicine) combined (10). Moreover, exercise therapists must deliver theoretical content and practical exercise sessions, posing specific challenges (e.g., media selection, digital learner support) in digital formats (11), potentially influenced by individual factors like age and gender (12). Beyond these general demands, exercise therapy also requires competences such as digitally demonstrating and correcting movement, adapting exercises to diverse physical abilities, and supporting self-regulated activity through digital monitoring and feedback. These go beyond the scope of general digital competence models, which rarely address motor learning, embodied interaction, or exercise-specific safety in digital settings (13). Addressing these needs requires theory-based, validated models to define competences required for digital obesity exercise therapy (DOET) (14). DOET is understood here, in line with the definition above, as the structured, digitally mediated delivery of exercise-based interventions comprising both physical activity and related educational or behavioral components, specifically targeting people living with obesity. Such competence models enable structured assessment, targeted training and curriculum development, supporting effective digital implementation; particularly for patients in structurally underserved regions.

However, no validated competence models exist for DOET. To address this gap, existing educational frameworks offer a valuable foundation for competence identification through conceptually and content-driven modeling approaches (15, 16). Accordingly, the internationally recognized DigCompEdu has attracted attention as transferable framework originally designed for educators but increasingly relevant for competence development in medical education. It defines digital competences across six dimensions: Professional Engagement, Digital Resources, Teaching and Learning, Assessment, Learning Strategy, and Facilitating Learners’ Digital Competence (17). DigCompEdu is distinguished by its dual methodology: target group input and empirical validation via latent construct analysis. In line with this, current best practices in validation begin with model-driven cognitive interviews to assess content validity. Subsequent factor analyses examine the factor structure and psychometric quality of the instrument (18). Through its methodological triangulation, DigCompEdu serves as diagnostic tool and transferable reference model in educational institutions for the use of digital resources ensuring broad applicability (19). This multi-stage validation approach is also reflected in more recent studies on digital competence, including those conducted in healthcare settings (20, 21).

Accordingly, transferring the DigCompEdu validation approach to DOET (22) requires reference to the only established competence model for exercise therapists in digital obesity therapy (DigCompThExO) by Pawellek and Wulff, with the questionnaire development process detailed elsewhere (23). Conceptually, DigCompThExO followed a two-step lineage: a) DigCompEdu as the overarching theoretical reference framework for digital competence development and b) domain-specific adaptation to the requirements of exercise therapy in digital obesity care. Unlike general digital health frameworks (24), DigCompThExO explicitly integrates didactic-motor aspects such as exercise demonstration, exercise feedback, and self-monitoring; elements largely absent from broader models. It addresses key therapeutic tasks including digital planning, motor instruction, behavior regulation, and patient-centered support in digitally mediated exercise contexts. Based on this foundation, DigCompThExO comprises five dimensions (Selection Criteria, Teaching Strategy, Learning Support, Media Reflection and Media Development) and is operationalized through an 18-item questionnaire. Competence is assessed on a six-point Likert scale, measuring two cognitive competence levels of knowledge and application. Thus, the DigCompThExO represents a novel instrument to identify competences to plan and implement DOET. It has potential to inform training programs, and guide professional development. However, adaptation and validation of the DigCompThExO's factor structure remain pending due to limited empirical groundwork, leaving its diagnostic utility unconfirmed.

Against this background, the central problem arises that, although a theory-based DOET competence questionnaire exists, its interpretive value depends on adaptation to the target group and statistical validation. Therefore, the present study pursued the following obejectives: 1. to examine whether DOET therapists interpret the questionnaire items as intended, thereby supporting content validity; 2. to test whether the hypothesized factor structure of DigCompThExO is confirmed through empirical data; and 3. to evaluate whether DigCompThExO demonstrates valid and reliable psychometric properties.

## 2 Materials and methods

To address these research questions, selected methods from the validation approach by Boateng and Neilands (18) were applied based on available data and study aims. Validation followed a two-step approach: qualitative cognitive interviews for questionnaire refinement (Study 1), followed by quantitative assessment of internal consistency, composite reliability, discriminant validity, and gender invariance (Study 2). All steps adhered to the Consensus-based Standards for the Selection of Health Measurement Instruments (COSMIN) guidelines for their specificity and international applicability (25).

### 2.1 Study 1: cognitive interviews

#### 2.1.1 Study Design

To assess content validity and identify comprehension issues, cognitive interviews were conducted with the target group (18), guided by the model of Tourangeau (26). Probing techniques (think-aloud, paraphrasing, process probing) addressed the cognitive stages of comprehension, retrieval, judgment, and response of items. Key foci included item clarity, relevance, and scale design. Data were collected between January and March 2024 in Germany.

#### 2.1.2 Participants

Following cognitive interview guidelines, a sample of five to 15 DOET therapists was defined to balance feasibility and comprehension coverage (27). Recruitment was conducted using a quota sampling approach, drawing from a convenience sample, with participants being contacted through exercise therapy institutions and sports clubs (28). Selection was guided by sociodemographic variables shown in literature to impact competence development (29): age was classified using the threshold proposed by Bachmann, Hertweck (30), distinguishing young and older adults (> 36 years) and gender (male and female).

#### 2.1.3 Administration

Interviews were conducted individually via Zoom by a trained interviewer to ensure procedural consistency. Following informed consent and a mandatory entry test (31), participants reviewed the questionnaire item by item (20–45 min) using a structured guide. All sessions were recorded for analysis.

#### 2.1.4 Data analysis

Interview data were transcribed via Whisper and analyzed using qualitative content analysis (32). A structured codebook guided the coding process, developed based on Tourangeau's cognitive interview framework and refined through peer debriefing with colleagues. Categories were derived deductively from the interview guide and relevant literature, and complemented inductively with data-driven codes. Although coding was conducted by a single researcher, peer debriefing supported methodological transparency and interpretive reliability. Modifications were made when ≥30% of therapists independently identified the same issue, indicating consensus. This threshold aligns with established practices in cognitive interviewing, where recurring issues identified by approximately one third of participants are considered indicative of systematic comprehension problems rather than individual idiosyncrasies (18, 31).

#### 2.1.5 Bias

Potential sources of bias include social desirability effects and interviewer-related assumptions, which may have caused confusion or influenced participants’ responses. Furthermore, recall bias cannot be fully excluded, given the retrospective nature of participants’ professional experiences.

### 2.2 Study 2: confirmatory factor analysis

#### 2.2.1 Study design

The cross-sectional study was designed to evaluate validity and reliability of the DigCompThExO. Data were collected between May 2024 and January 2025. In addition to competence-related responses, participants provided demographic information (age, gender).

#### 2.2.2 Participants

For statistical validation, a 10:1 respondent-to-item ratio required a minimum of 180 participants based on the DigCompThExO's 18 items; to ensure power and account for exclusions, a target of 200–300 responses was set (33, 34). In line with psychometric guidelines for confirmatory factor analysis, this target range is considered adequate for models of moderate complexity and acceptable item quality (35), particularly given the DigCompThExO's focused four-factor structure and theoretical foundation. Eligible participants were certified exercise therapists (degree, vocational training, or other) with experience in obesity care, and use of any media in therapy. Eligibility was verified using professional background data (e.g., qualification, experience) according to Moosbrugger and Kelava (14). Therapists were recruited via email from therapy and rehabilitation centers in Germany, Austria, and Switzerland, partly identified through listings of certified centers by the German Obesity Society.

#### 2.2.3 Administration

DigCompThExO was administered anonymously online via LimeSurvey. Participants were informed about study objectives, confidentiality, voluntary participation, and lack of financial incentives. Response formats were explained in advance; completion took approximately ten minutes. While participation was recommended via laptop for optimal graphical display, the survey was also accessible on phones and tablets.

#### 2.2.4 Statistical analysis

To assess the structural and construct validity of the DigCompThExO, a Confirmatory Factor Analysis (CFA) was conducted in R (Version 4.4.2) using the lavaan package (36). Following Boateng et al.'s framework (18), CFA was chosen over Exploratory Factor Analysis (EFA) due to the instrument's strong theoretical foundation, predefined structure, and limited item count (18 items), which rendered EFA inappropriate given the low item-to-factor ratio and the associated risk of model instability (35, 37, 38). Model fit was evaluated using the Comparative Fit Index (CFI), Tucker–Lewis Index (TLI), Root Mean Square Error of Approximation (RMSEA), Standardized Root Mean Square Residual (SRMR), and the chi-square statistic (*χ*²/df). Thresholds for acceptable model fit were set at CFI and TLI  > .95, and RMSEA and SRMR < .08, in line with recommendations by literature (39). While Hu and Bentler (39) suggest a stricter RMSEA cutoff of .06 for good fit, values up to .08 are commonly considered acceptable (40), particularly in models of moderate complexity and sample size. Thresholds for acceptable fit were CFI and TLI > .95, RMSEA and SRMR < .08. Internal consistency was assessed via Composite Reliability (CR ≥ .70 acceptable), which offers improved accuracy over Cronbach's alpha for digital competence constructs (41). Convergent validity was tested using Average Variance Extracted (AVE > .50), construct validity through correlation analysis (r > .50), and discriminant validity via the Heterotrait-Monotrait Ratio (HTMT < .85) (42, 43). Gender invariance was examined following Vandenberg and Lance (44) using RMSEA ≤ .08 as an indicator of acceptable model fit.

#### 2.2.5 Missing data

Incomplete questionnaires were excluded, rendering missing data handling unnecessary.

#### 2.2.6 Post-hoc analysis

*post-hoc* model modifications followed the planned validation strategy, guided by modification indices and theoretical coherence. As recommended in CFA, adjustments were only made when theoretically justified, and were clearly reported and cross-validated to ensure robustness (37).

#### 2.2.7 Bias

Potential biases may stem from convenience and self-selection, as therapists were recruited via email from certified centers in German-speaking countries. This likely favored younger and more digitally engaged professionals, limiting generalizability to older therapists or those in less connected or international settings. Future studies should use broader sampling strategies to validate subgroup differences.

## 3 Results

### 3.1 Study 1: cognitive interviews

Ten out of 13 invited exercise therapists from Leipzig participated in the interviews (Age: M = 39.3, SD = 10.7, 50% male). Participants had prior experience working with patients affected by obesity.

Findings from the interviews indicated overall alignment with intended item meanings, though minor interpretational differences emerged. Based on Tourangeau's (26) four cognitive stages of item response (comprehension, retrieval, judgment, and response), areas for improvement were identified. The following sections provide a detailed breakdown of each stage.

#### 3.1.1 Comprehension

Participants showed adequate comprehension of the items and could paraphrase them accurately. However, four participants misinterpreted “digital media”, referring only to hardware rather than both hardware and software, an issue further complicated by the term “digital resources” in the Media Development dimension. For example, one participant stated: “When I read digital media, I mainly think of devices like tablets or cameras, not software applications.”

#### 3.1.2 Retrieval

Most participants retrieved relevant information quickly, though five therapists needed reflection for soft- and hardware development examples. Two also struggled to recall data protection strategies. Six therapists were unsure whether the item referred to feedback from therapists to participants or vice versa regarding media use: “I wasn’t sure whether the item refers to my feedback to patients or feedback I receive from them.” This quotation illustrates recurring comprehension ambiguities that guided item revision.

#### 3.1.3 Judgment

While the overall structure of the questionnaire was perceived as intuitive, participants’ ability to judge the relevance and applicability of Media Development dimension revealed uncertainty. Specifically, questions about hardware development were deemed irrelevant to therapeutic work: “I mean, I studied sport science, not computer science.” Five participants raised concerns about software development, though three acknowledged their competence in software adaptation after further reflection.

#### 3.1.4 Response

The division of response options into knowledge (“know it”) and application (“use it”) caused confusion. While the six-point Likert scale for application was perceived as clear, seven therapists viewed knowledge as a dichotomous trait, making its assessment complex. Moreover, they noted that knowledge is a prerequisite for application, rendering its separate measurement partially redundant.

#### 3.1.5 Revisions

The following revisions to the questionnaire were made based on the key cognitive errors:

**Clarity**: The questionnaire's introduction was adjusted to emphasize that digital media includes both hardware and software, and all related terms (e.g., digital resources) were standardized accordingly. The direction of the feedback was clarified, to enhance recipients’ understanding.

**Relevance**: Questions regarding hardware development were reduced, so that the Media Development dimension was limited to software development.

**Scale units**: In the revision process, the original knowledge items (“Know it”) were removed due to limited discriminatory power and high overlap with the corresponding application items, resulting in conceptual redundancy and reduced psychometric utility.

The revised dimensions and items are presented in Table 1.

**Table 1:** The DigCompThExO dimensions and included items following cognitive interviews.

Dimension	Question	Item	Item abbrev.
Selection Criteria (SC)	I use Selection Criteria for…	Software in my DOET area.	SC1
		Hardware in my DOET area.	SC2
		The protection of personal data.	SC3
Teaching Strategy (TS)	I use digital media for…	The individual activation of participants.	TS1
		The demonstration of practical DOET content.	TS2
		The explanation of theoretical DOET content.	TS3
		Providing feedback to participants.	TS4
Learning Support (LS)	I use digital media that enable participants to…	Plan their DOET-related behavior.	LS1
		Document their DOET-related behavior.	LS2
		Monitor their DOET-related behavior.	LS3
		communicate.	LS4
Media Reflection (MR)	I reflect before each session with digital media on…	Why I use them in DOET.	MR1
		How I use them in DOET.	MR2
		When I use them in DOET.	MR3
		For whom I use them in DOET.	MR4
Media Development (MD)	I can…	Create software.	MD1
		Adapt software.	MD2
		Combine different software.	MD3

DOET, digital obesity exercise therapy; digital media, hardware and software.

This content validation provides the basis for subsequent statistical validation (CFA) to develop a standardized diagnostic tool and address the identified research gap.

### 3.2 Study 2: confirmatory factor analysis

The online study conducted via LimeSurvey yielded a total of 246 collected questionnaires. After excluding incomplete responses, 205 complete questionnaires were analyzed. The separate sample, independent of the cognitive interview participants, had a mean age of 32.45 years (SD = 5.9). The gender distribution included 52.2% men (n = 107), 47.3% women (n = 97), and 0.5% diverse (*n* = 1). The majority of participants were of German nationality (88.8%, *n* = 182), followed by Austrian (8.3%, *n* = 17), Dutch and Polish (1.0% each, *n* = 2), Italian and Swiss (0.5% each, *n* = 1).

The assumptions for confirmatory factor analysis were met, and despite non-normal data distribution, the MLR estimator provided robust parameter estimates. The initial five-dimensional model (M0) showed a moderate fit [*χ*²(125) = 351.650; *p* < .001; CFI = .917; TLI = .898; RMSEA = .086, 90% CI [.073;.100]; SRMR = .087]. Anomalies were observed with Item MD2, including a factor loading > 1 and a negative variance (.073), indicating a Heywood case. In addition to this statistical irregularity, the cognitive interviews also revealed issues related to the Media Development dimension, as therapists did not consider software creation or further development to fall within their therapeutic scope of practice. Therefore, Media Development dimension was excluded from further analyses.

An adjusted four-dimensional model (M1) showed improved fit [*χ*²(84) = 172.013; *p* < .001; CFI = .944; TLI = .929; RMSEA = .083, 90% CI [.065;.101]; SRMR = .074]. As the RMSEA exceeded the.08 threshold, correlations between dimensions were examined. A high covariance between Selection Criteria and Teaching Strategy (Cov = .952; *p* > .001) indicated conceptual overlap. Beyond statistical fit, their merging was theoretically supported by the close functional link between media selection and instructional planning, as emphasized in media didactics (45) and outlined in the introduction. The combined dimension (Media Application Strategy) reflects this integrated decision-making process.

The three-dimensional model (M2) showed good fit [*χ*²(87) = 181.672; *p* < .001; CFI = .939; TLI = .926; RMSEA = .085, 90% CI [.060;.102]; SRMR = .076]. However, a Likelihood Ratio Test revealed that the four-dimensional model [*χ*²(84) = 231.52] fit the data significantly better than the three-dimensional model [*χ*²(87) = 245.75; *Δχ*² = 9.23; *p* = .026].

Yet, to improve fit of the four-dimensional model, modification indices were calculated following Saris, Satorra (46), considering only values exceeding > 10, as these indicate a statistically significant improvement in model fit [*p* < .01; *χ*²(1) improvement] while minimizing the risk of overfitting (see Table 2) (47).

**Table 2:** Modification indices for optimizing the four-dimensional model.

Dimension	Item	MI
Selection Criteria (SC)	LS4	44.865
	TS1	16.662
	LS2	11.923
	MR4	10.511
Teaching Strategy (TS)	LS4	44.875
	MR4	19.167
	MR3	18.597
	LS2	10.834
Learning Support (LS)	MR4	25.168
	TS1	15.395
	MR3	11.720
Media Reflection (MR)	LS4	39.964
	LS2	10.179

MI, modification indices.

**Figure 1:**
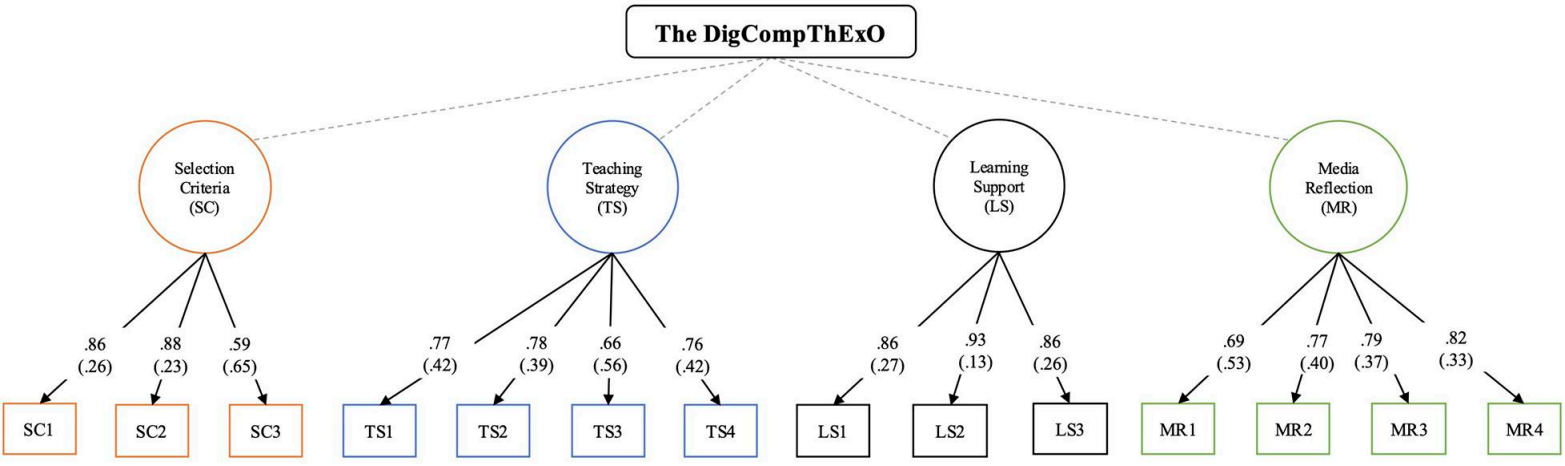
The final DigCompThExO model.

Item LS4 (digital communication), which cross-loaded all factors, was identified as a key source of misfit. As communication is conceptualized as a prerequisite for learning rather than a distinct competence (48), the item was removed and the model was re-estimated. The revised four-factor model (M3) showed improved fit: CFI = .964; TLI = .953; RMSEA = .070, 90% CI [.049;.090] and SRMR = .051. Standardized factor loadings for all 14 items ranged from 0.59 to 0.93 (M = 0.78), confirming strong item-factor relationships. A full list of loadings is provided in Appendix A. The final model is shown in Figure 1. Values on arrows represent standardized factor loadings; residual variances are shown in parentheses.

To evaluate the impact of model modifications, the M3-model was compared to the previous M1-model using a Likelihood Ratio Test with fit assessed via the Akaike Information Criterion (AIC) and Bayesian Information Criterion (BIC). The M1-model had an AIC of 7,695.1 and a BIC of 7,814.7, while the M3-model showed improved fit (AIC = 7,100.6; BIC = 7,213.6), indicating a better modeling of the construct. Table 3 summarizes all models and their fit indices in the order of modifications made during the research process.

**Table 3:** Summary of calculated models and their fit indices.

Model	Code	*χ* ^2^	df	*p*	CFI	TLI	RMSEA [90%CI]	SRMR
5-factor model	M0	351.650	125	<.001	.917	.898	.086 [.073;.100]	.087
4-factor model	M1	172.013	84	<.001	.944	.929	.083 [.065;.101]	.074
3-factor model	M2	181.672	87	<.001	.939	.926	.085 [.060;.102]	.076
4-factor model without LS4	M3	124.289	71	<.001	.964	.953	0.70 [.049;.090]	.051

*χ*^2^, chi-square statistic; CFI, comparative fit index; TLI, Tucker–Lewis index; RMSEA, root mean square of approximation; SRMR, standardized root mean square residual.

Convergent validity was supported by AVE values above.50 (SC = .62; TS = .55; LS = .78; MR = .59), indicating adequate variance explanation. Internal consistency was confirmed by CR values exceeding.70 (SC = .83; TS = .83; LS = .91; MR = .85).

To ensure minimal interdimension correlation, discriminant validity was assessed using the Heterotrait-Monotrait ratio (HTMT). All HTMT values were below the threshold of.85, confirming adequate discriminant validity between the dimensions of Selection Criteria (SC), Teaching Strategy (TS), Learning Support (LS), and Media Reflection (MR) (SC-TS = .79; SC-LS = .62; SC-MR = .77; TS-LS = .71; TS-MR = .75; LS-MR = .54).

The questionnaire revealed a clear four-factor structure with 14 items and strong construct validity. Measurement invariance across gender was tested using configural (I0), metric (I1), and scalar (I2) models. Due to the limited sample of diverse participants (*n* = 1), only male and female groups were analyzed. The configural model (I0) showed an acceptable fit [RMSEA = .074; 90% CI (.051;.096)], providing a baseline. The metric model (I1) indicated good fit [RMSEA = .069; 90% CI (.045;.091)], supporting equivalence in factor loadings. The scalar model (I2) also showed good fit, with the same RMSEA value, 90% CI [.046;.090], confirming scalar invariance across gender. Since model comparisons revealed no substantial loss in fit (*Δ*CFI ≤ .005; *Δ*RMSEA = .000), full scalar invariance was established, and partial invariance testing was not required. These results suggest equivalent construct measurement across genders (compare Table 4).

**Table 4:** Measurement invariance across gender.

Model	Df	AIC	BIC	*χ* ^2^	*Δχ* ^2^	*Δ*df	*p(Δχ*^2^)
I0	142	7,108.3	7,426.8	256.16			
I1	152	7,096.2	7,381.6	264.13	6.0214	10	.8135
I2	162	7,090.9	7,343.0	278.76	13.2281	10	.1628

l0, configural model; l1, metric model; l2, scalar model; Df, degrees of freedom; AIC, akaike information criterion; BIC, bayesian information criterion; *χ*^2^, chi-square statistic; *Δχ*^2^, difference in chi-square statistics; *Δ*df, difference in degrees of freedom; *p*(*Δχ*^2^), *p*-value for chi-square difference.

## 4 Discussion

To tackle the challenges of digital transformation, this study validated the theory-based DigCompThExO questionnaire to assess digital competences of exercise therapists, guided by three research questions. First, cognitive interviews confirmed item clarity, relevance, and target-group appropriateness; resulting revisions (e.g., clarifying terminology, response simplification) improved content validity. Second, confirmatory factor analysis supported the hypothesized structure, with the revised model demonstrating good fit and thereby evidencing structural validity. Third, psychometric testing confirmed reliability and convergent validity. These findings establish the DigCompThExO as a diagnostic tool for competence development in DOET and contribute to advancing digital transformation within medical education and therapeutic practice. In practice, DigCompThExO can be used as a diagnostic entry tool in academic curricula and continuing professional development to identify individual competence profiles. Based on these profiles, modular training formats (e.g., workshops on digital feedback or self-monitoring tools) can be tailored to therapists’ specific developmental needs.

### 4.1 Interpretation of results

Due to limited data on exercise therapists in digital obesity therapy, professions with comparable implementation tasks (e.g., psychotherapy, physiotherapy) and digitally competent educators from non-medical contexts (comparable knowledge transfer) were included to underpin four key discussion points on the DigCompThExO's revision.

First, the DigCompThExO was revised based on cognitive interviews and statistical analyses, leading to the removal of the Media Development dimension due to its limited perceived relevance. Similar challenges have been reported in physiotherapy, a discipline grounded in exercise and practical interaction. In this field, limited media development is linked to the disconnect between pedagogical technology use and therapeutic practice (49, 50). Comparable issues arise in digital instruction within educational institutions, where the focus has shifted towards effective use and pedagogical adaptation of existing tools, rather than creating new media (51, 52). Despite this shift, DigCompEdu promotes digital content creation, assuming that educators (or therapists) should act as media designers to support individual learning (17, 53). While it remains unclear whether such expectations are realistic in therapeutic settings, growing demands for media development in exercise-related fields suggest relevance. This is particularly for true for the context examined here, where individualized digital tools are central to behavioral change (54). Consequently, given current challenges in adapting technology to individual needs, future studies should explore user acceptance and pilot selected media in RCTs to evaluate feasibility, efficacy and impact. Such evidence is crucial before recommending extensive media-related training, especially given the highly diverse ways digital media are used in therapy.

Second, reduction of the communication item was necessary due to poor model fit, highlighting persistent ambiguity around its role in digital education as well as therapy. While DigCompEdu defines communication as a core competence for structuring group interactions and learner engagement (17, 55, 56), therapeutic communication relies on nuanced, interpersonal exchanges within one-on-one or small-group interactions (48, 57, 58). Digital formats disrupts this by limiting nonverbal cues and tactile feedback (59, 60), forcing therapists to verbalize even implicit aspects of movement and interaction (61). Beyond interpersonal aspects, digital therapy also demands technical-exploratory communication, such as clarifying the purpose and function of digital tools (62). Yet, DOET therapists examined here struggled even to define “digital resources”, revealing striking conceptual uncertainty. Also, it is unresolved whether digital competence is a perquisite for digital communication or vice versa (63). Consequently, existing competence models may insufficiently reflect the specific communicative demands of digital therapy. Future digitalization efforts should define key conditions (e.g., group size, interaction level) and embed them in didactic design to compensate for the lack of physical presence. Only then can training address both empathic and technical dimensions of digital communication.

Third, the six-point Likert scale was reduced to competence application, as knowledge aspects were deemed overly complex and unclear during cognitive interviews. This marks a deliberate shift from assigning competences to broad cognitive stages towards capturing their nuanced expression within a single process via metrically structured scaling. This approach contrasts with the widely adopted DigCompEdu (64, 65). While enabling self-placement, it lacks psychometric rigor due to undefined level intervals and limited granularity; constraining statistical comparability and precise measurement of competence progression (17, 66). In contrast, the Likert scale used here enables fine-grained profiling of applied competences, supports metric approximation and is well suited to heterogeneous therapeutic settings (67), especially in addressing digital competences (68, 69). Ideally, each nuanced expression of competence across cognitive levels would be captured; however, such granularity places respondent burden (70). Consequently, this highlights the need for empirical studies to evaluate the cost-benefit trade-off, thereby informing evidence-based decisions regarding both the design and application of questionnaires. To ensure systemic alignment, these decisions should be developed in dialogue with key stakeholders (e.g., therapy providers, associations).

Fourth, contextual delimitation of competence frameworks is critical for valid interpretation and use. DigCompThExO's streamlined, four-dimensional structure (14 items) aligns with physical education instruments prioritizing parsimony and feasibility over item volume (71). This approach supports cognitive efficiency and reduced respondent burden without compromising content validity (72). In contrast, educational models such as DigCompEdu have expanded to include contextual dimensions (e.g., digital environment, extrinsic digital engagement), reflecting the resource-bound nature of education-based practice (73). Comparable relevance has been shown in healthcare, where digital competence is shaped by systemic factors, prompting dual-instrument models to capture both skills and enabling conditions (24, 74). Similar dependencies may also affect DOET therapists examined here, whose competence is shaped by institutional and national-level constraints (e.g., choice or funding for digital infrastructure) (75). Consequently, future extensions of DigCompThExO should incorporate context-related dimensions and stakeholder perspectives to clarify decision-making processes around digital infrastructure in relation to cost-benefit considerations. When measurable, contextual factors (e.g., digital infrastructure) could be examined through statistical analysis to explore their influence on therapists’ competence development, thereby informing more targeted and setting-specific training strategies.

### 4.2 Strengths and limitations

As a cross-sectional self-report questionnaire, the DigComThExO is inherently susceptible to biases such as recall errors, social desirability, and interviewer influence; potentially limiting the accuracy and objectivity of competence ratings. Standardized, experience-based interviews were used to mitigate these effects; however, future studies should incorporate objective measures (e.g., observation, third-party ratings).

Furthermore, DigCompThExO may be applied longitudinally to track competence development over time, for example before and after targeted training interventions or during professional socialization. In the present study, the relatively young, self-selected sample may have led to a systematic overrepresentation of digitally confident participants, potentially inflating overall competence scores and limiting generalizability to older or less digitally experienced therapists.

Potential item bias by gender or professional experience also cannot be ruled out. Future studies should examine differential item functioning to ensure measurement equivalence across subgroups. Such bias could affect group comparisons and obscure true competence differences.

Another limitation is the lack of test-retest reliability assessment, which prevents conclusions about the temporal stability of the scale and the reproducibility of individual competence scores. This was not feasible due to several constraints, including the cross-sectional study design, the sample size required for longitudinal follow-up, and the professional context of participants, which limited the feasibility of repeated measurement. However, measurement invariance across gender supports structural consistency. Future studies should implement longitudinal or repeated-measures designs (e.g., pre-post evaluations of trainings) to assess stability over time.

The removal of the knowledge-level items (“Know it”) during scale revision represents an additional limitation. This decision was based on high empirical overlap with the application items and participant feedback from cognitive interviews, where the distinction was perceived as redundant and burdensome in clinical routines. While this improved feasibility and focus, it may reduce conceptual differentiation between knowing and applying digital competences. Future studies could explore more efficient ways to capture knowledge aspects.

Finally, the questionnaire was developed and validated in a German-speaking sample. For cross-national and broader international applicability, translation and cultural adaptation following established guidelines (e.g., forward-back translation, cognitive pretesting) are required. Subsequent validation should confirm construct validity and measurement invariance across cultural or institutional contexts. This is essential to ensure generalizability and contextual relevance across health systems.

Despite limitations, this study offers key strengths. Methodological triangulation across qualitative and quantitative data ensured comprehensive construct validation. CFA further confirmed structural robustness in handling complex datasets. Finally, COSMIN-aligned methods ensured validation rigor.

## 5 Conclusion

DigCompThExO provides foundation for identifying existing competences and developmental needs, assuming future pilot applications enable the identification of current competence profiles among DOET therapists. Deriving from this, training programs should be developed and evaluated through RCTs, assessing competence acquisition and subsequent therapeutic outcomes. If effective, integration into academic curricula and continuing education for DOET professionals is imperative to ensure consistent qualification standards. Such integration may further support the establishment of accreditation criteria for DOET, enabling regulatory bodies and professional associations to align certification, licensing requirements, and reimbursement structures with validated competence standards. Only through trained professionals can DOET advance from pioneering interventions, such as the German KLAKS^online^ program (8), into a scalable, evidence-based standard of care. At the policy level, the instrument offers a foundation for defining minimal digital competence standards in national treatment guidelines and quality assurance frameworks. This could inform strategic workforce planning, support reimbursement negotiations for digital services, and guide the development of accreditation pathways for DOET providers. However, strategies for integrating the questionnaire into practice and embedding derived training into structured education remain to be determined.

Beyond its immediate use, DigCompThExO offers a methodological and conceptual blueprint for future research. Cross-cultural adaptation may enable valid assessment of exercise therapist competences beyond German-speaking countries. This presupposes that the previously discussed challenges, such as media effects, digital communication, and contextual influences, are adequately addressed. Comparative insights could inform international training strategies. Additionally, given the multidisciplinary nature of obesity therapy, the tool may be tailored to other obesity therapy professionals (nutritionists, psychologists) to support discipline-specific competence training. Beyond obesity-specific contexts, the questionnaire may be applied in broader rehabilitation settings and in the treatment of other chronic conditions (e.g., asthma, diabetes). It also holds potential for use in physiotherapy, sport science, and health promotion, where digital interventions and emerging technologies such as artificial intelligence are gaining importance.

In summary, this study closes a critical gap in the assessment of digital competences in obesity exercise therapy by introducing the DigCompThExO, the first validated instrument in DOET. Such questionnaires are essential to meet the evolving competence demands, where exercise therapists must translate obesity-specific content into digital formats across technical, pedagogical, and therapeutic domains. However, how the questionnaire should be implemented and which requirements are needed to derive effective training programs remains unclear, particularly in the light of the unsolved research questions outlined above. If these questions are systematically addressed, the study lays the groundwork to support the integration of digital competencies into medical education and promote more equitable digital access to obesity therapy.

## Data Availability

The raw data supporting the conclusions of this article will be made available by the authors, without undue reservation.

## Appendix A: Standardized factor loadings (Std.all) for the final 14-item, 4-factor model.

**Table:**

Factor	Item	Loading (Std.all)
Selection Criteria (SC)	SC1	0.862
	SC2	0.878
	SC3	0.592
Teaching Strategy (TS)	TS1	0.765
	TS2	0.779
	TS3	0.661
	TS4	0.760
Learning Support (LS)	LS1	0.858
	LS2	0.931
	LS3	0.862
Media Reflection (MR)	MR1	0.686
	MR2	0.772
	MR3	0.794
	MR4	0.820

Std.all, standardized factor loadings.
